# COVID-19 Score for Testing Symptomatic Low Risk Children: “STUDY SAFE”

**DOI:** 10.3389/fmed.2021.644813

**Published:** 2021-06-15

**Authors:** Gavriela Feketea, Vasiliki Vlacha

**Affiliations:** ^1^“Iuliu Hatieganu” University of Medicine and Pharmacy, Cluj-Napoca, Romania; ^2^Paediatric Department, Karamandaneio Children's Hospital of Patras, Patras, Greece; ^3^Department of Early Years Learning and Care, University of Ioannina, Ioannina, Greece

**Keywords:** RT-PCR—polymerase chain reaction with reverse transcription, SARS-CoV-2, COVID-19, scoring—algorithm, safety

Telemedicine has undergone remarkable expansion since the beginning the COVID-19 pandemic (1), for visits related not only to COVID-19, but also other acute and chronic illnesses. The major advantage of telemedicine is the opportunity for patients to receive medical advice in their own home, without the risk of exposure to SARS-CoV-2. It has been shown that virtual medical visits significantly decrease the mortality of some medical conditions such as cardiac diseases (2). The use of algorithms appears to facilitate the practice of telemedicine.

SARS-CoV-2 has expanded rapidly over the world. The vaccines against COVID-19 have been implemented extensively by many countries but herd immunity is, as yet, far from being achieved. Currently, only one vaccine has been authorized to be used for adolescents above the age of 16 years. However, according to the recommendations of the US Centers for Disease Control and Prevention (CDC), even if a youngster has been immunized, testing should be done in the case of symptoms related to COVID-19 (3). Most of the children infected with SARS-CoV-2 are asymptomatic or develop mild symptoms, but even in the presymptomatic or asymptomatic state, school-age children and adolescents can transmit the infection to vulnerable individuals.

During the past winter, 2020–2021, a significant decrease in the prevalence of respiratory viral infections was noted, which is attributed to the preventive measures instituted against COVID-19 (4). As a result, children with viral symptoms are regarded as having a high suspicion of COVID-19 infection. Both the CDC and the European Centre for Disease Prevention and Control (ECDC) recommend the testing by RT-PCR for SARS-CoV-2 of all patients presenting to the health care system with symptoms compatible with COVID-19 infection, as part of active case finding for SARS-CoV-2 (5, 6), wherever testing capacity is sufficient. Testing all children with fever and/or cough will be difficult for several countries with limited test supplies and/or when the health system is overwhelmed. In an attempt to conserve resources and to formulate the means of evaluation via telemedicine, taking into consideration the evidence that children usually suffer from mild COVID-19 infection, we developed the current “STUDY SAFE” algorithm.

This scoring system is straightforward and is based on epidemiological data concerning the child's exposure risk for COVID-19 (Figure 1). It can be easily implemented in telemedicine consultations, and can guide primary care physicians in making a decision about when a diagnostic RT-PCR test for COVID-19 needs to be performed in symptomatic school age children. The diagnostic tests for COVID-19 can be conducted at home by the caregiver, under the virtual guidance of the primary care doctor.

**Figure 1:**
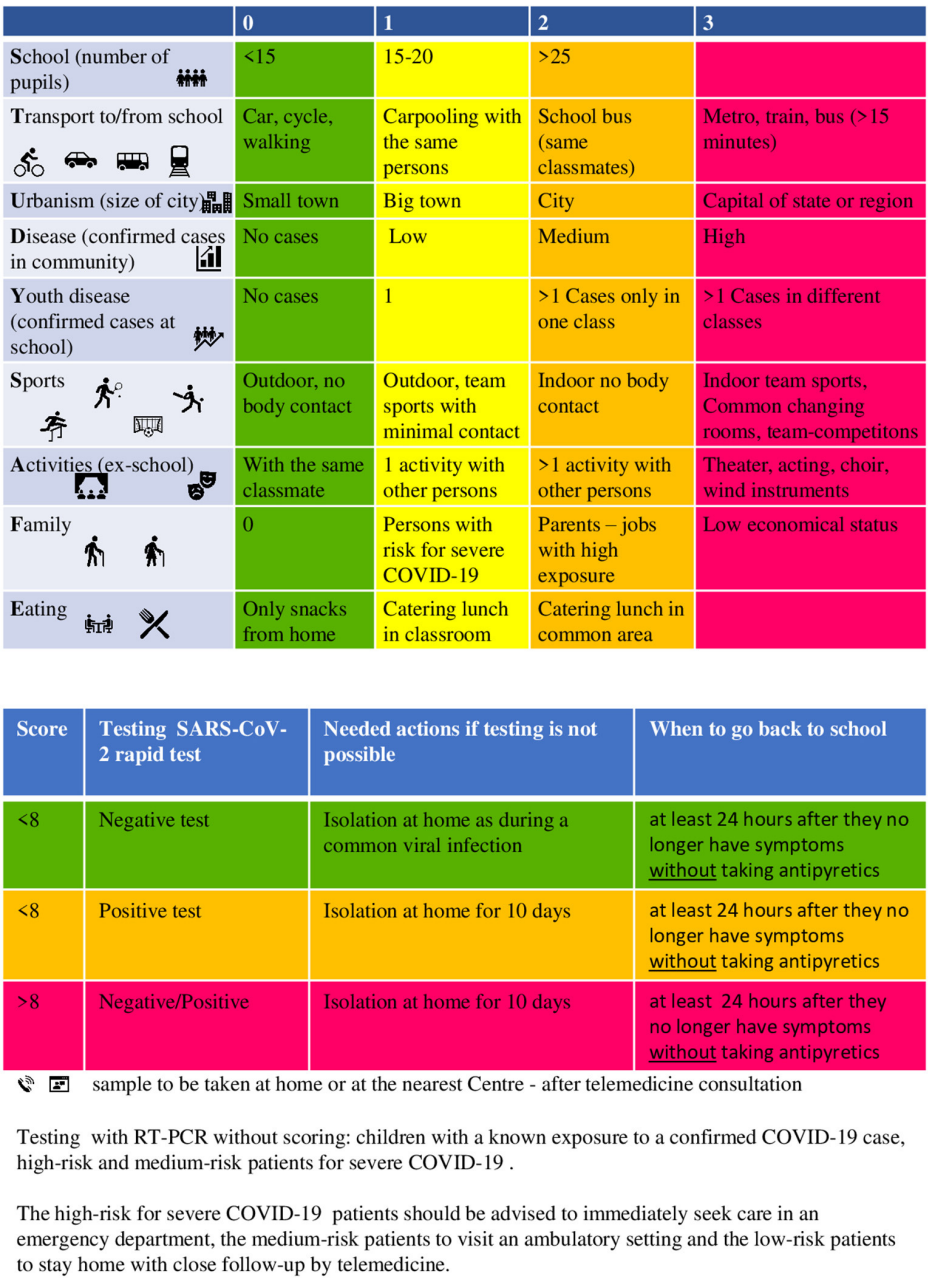
COVID-19 score “STUDY SAFE” for testing low-risk school-age children with symptoms compatible with COVID-19.

The patients at high-risk for severe COVID-19, such as children with comorbidities, and those with known exposure, will undergo testing without the need for scoring according to the algorithm. In addition, the children with moderate or severe symptoms should be advised to seek medical care immediately in an emergency department or an ambulatory health care setting, according to the severity of the symptoms (7); prior notification of the health care department is highly recommended.

The score provided by the algorithm is calculated based on the risk of contracting and/or transmitting SARS-CoV-2. Various factors are taken into consideration, including the socioeconomic status, the risk of exposure, based on the nature of the child's activities, the practice of social distancing and the spread of SARS-CoV-2 in the local community.

The criteria for scoring stratification were determined based on the current knowledge and recommendations of public health authorities with regard to the risk for SARS-CoV-2 transmission in nine contexts, including the population density of the area, confirmed cases in the community and at school, the child's age, the number of pupils per school class, mode of transport, sports, extracurricular activities, family setting, dining conditions.

All the children with symptoms related to COVID-19 should remain at home and be tested with the SARS-CoV-2 rapid test. The children with a low score (<8) remain at home until their symptoms are improved and they have been fever free for 24 h without antipyretics. They can come out of confinement 24 h after they become afebrile without antipyretics and the rest of the symptoms have improved, according to CDC guidelines (8). The children with a positive rapid test should remain in isolation at home for 10 days. The children with a score of above 8 should remain in isolation for 10 days, even in the case of a negative rapid test. We came to that decision based on fact that this group of children have extensive social interactions, and the sensitivity of the SARS-CoV-2 rapid test has been reported to be between 34 and 88% (9). In order to avoid the diverse outcome of false negative results in those children with many possible contacts, we suggested that they remain at home for 10 days. They can come out of isolation when they have been afebrile for 24 h without antipyretics and the rest of their symptoms are improved.

The World Health Organization (WHO) recommends that schoolchildren maintain a physical distance of at least 1 m inside the classrooms and in areas with community transmission. In the case of cluster-transmission, WHO recommends a risk-based approach, and in areas with sporadic cases/no cases of COVID-19, only the children aged above 12 years should keep the physical distance of 1 m (10). According to the CDC, children in larger in-person activities and events have a medium risk for SARS-Co-2 transmission (5). We consider low risk for COVID-19 transmission those classrooms with <15 pupils and high risk those with more than 25 pupils.

There is increasing evidence that indoor sports and sports with intensive body contact pose a higher risk of transmission than outdoor athletics and non-contact sports. CDC categorize team-based practice as increased risk, and as higher risk full competition between teams (5).

It has been shown up to the present, that the older children are responsible for higher transmission rate of SARS-CoV-2. The infection rate among family members in Southern Korea from index cases aged 0–9 years and 10–19 years was 5.3 and 18.6%, respectively (11). Based on that evidence we placed children aged above 10 years in a higher risk group for transmission.

With regard to dining at school, the CDC recommends the children to have meals outdoors as much as possible. When this is not feasible, they advise maintenance of a distance of 6 feet (2 m) while eating or in the food service line (5). In the present risk assessment, we assigned higher points when the children were eating in the cafeteria compared with having lunch in the classroom.

During school transit, CDC recommends preferring forms of transport with minimal contact (5). In this algorithm we placed the children at higher risk for transmision when they were using public transport or the school bus for a trip of longer than 15 min.

The confirmed cases in the community are considered as “Low” when <20 new cases have been diagnosed per 100,000 people, “Medium” when 20–59.9/100,000 people have been diagnosed and “High” when >60/100,000 have been diagnosed in the past 2 weeks (12).

Every child with symptoms related to COVID-19 should have a SARS-CoV-2 rapid self-test performed by their parents. In Greece, the self-tests were introduced for surveillance in school-age children on a weekly basis in April 2021. In other countries, including the UK and Germany, such tests have been available for school children since February 2021.

The scoring system presented here could be a useful tool for screening school age children without comorbidities, presenting with mild symptoms, for performing RT-PCR test for SARS-CoV-2. The goal is to prevent COVID-19 transmission in the community. After the successful “stay home, stay safe” policy, as the schools are reopened, the school children will still need to stay at home when they are sick, preventing the viral spread, and return to school and their other activities when the risk of transmission has passed. As more information becomes available about COVID-19, this “STUDY SAFE” score may need revisions, but it is a starting point for clinicians to manage children without comorbidities in COVID-19 era. The diagnostic test for COVID-19 can be done at home by the parent under the virtual guidance of the clinician. The algorithm presented is a simple tool to guide the physicians and can be easily performed via telemedicine.

## Author Contributions

GF and VV contributed equally to the design and implementation of the research, to the analysis of the results, to assemble the figure, and to the writing of the manuscript. All authors contributed to the article and approved the submitted version.

## Conflict of Interest

The authors declare that the research was conducted in the absence of any commercial or financial relationships that could be construed as a potential conflict of interest.

## References

[1] Ye S Kronish I Fleck E Fleischut P Homma S Masini D. Telemedicine expansion during the COVID-19 pandemic and the potential for technology-driven disparities. J Gen Intern Med. (2021) 36:256–8. 10.1007/s11606-020-06322-y 33105000 PMC7586868

[2] Lange SJ Ritchey MD Goodman AB Dias T Twentyman E Fuld J. Potential indirect effects of the COVID-19 pandemic on use of emergency departments for acute life-threatening conditions — United States, January–May 2020. MMWR Morb Mortal Wkly Rep. (2020) 69:795–800. 10.15585/mmwr.mm6925e2 32584802 PMC7316316

[3] Christie A Mbaeyi SA Walensky RP. CDC Interim Recommendations for fully vaccinated people: an important first step. JAMA. (2021) 325:1501–2. 10.1001/jama.2021.4367 33688914

[4] Chiu NC Chi H Tai YL Peng CC Tseng CY Chen CC. Impact of wearing masks, hand hygiene, and social distancing on influenza, enterovirus, and all-cause pneumonia during the coronavirus pandemic: retrospective national epidemiological surveillance study. J Med Internet Res. (2020) 22:e21257. 10.2196/21257 32750008 PMC7471891

[5] US Centers for Disease Control and Prevention. Coronavirus Disease 2019 (COVID-19). Available online at: https://www.cdc.gov/coronavirus/2019-ncov/community/schools-childcare/index.html (accessed September 25, 2020).

[6] European Centre for Disease Prevention and Control. COVID-19 Testing Strategies and Objectives. Available online at: https://www.ecdc.europa.eu/en/publications-data/covid-19-testing-strategies-and-objectives (accessed September 25, 2020).

[7] Feketea GM Vlacha V. A decision-making algorithm for children with suspected coronavirus disease 2019. JAMA Pediatrics. (2020) 174:1220–2. 10.1001/jamapediatrics.2020.2999 32955559

[8] US Centers for Disease Control and Prevention. When You Can be Around Others After You Had or Likely Had COVID-19. Available online at: https://www.cdc.gov/coronavirus/2019-ncov/if-you-are-sick/end-home-isolation.html (accessed May 7, 2021).

[9] Griffin S. Covid-19: Lateral flow tests are better at identifying people with symptoms, finds Cochrane review. BMJ. (2021) 372:n823. 10.1136/bmj.n823 33766893

[10] World Health Organization. Q&A: Schools and COVID-19. Available online at: https://www.who.int/emergencies/diseases/novel-coronavirus-2019/question-and-answers-hub/q-a-detail/q-a-schools-and-covid-19 (accessed September 25, 2020).

[11] Park YJ Choe YJ Park O Park S Kim YM Kim J. Contact tracing during coronavirus disease outbreak, South Korea, 2020. Emerg Infect Dis. (2020) 26:2465–8. 10.3201/eid2610.201315 32673193 PMC7510731

[12] European Centre for Disease Prevention and Control. Coronavirus Disease 2019 (COVID-19) in the EU/EEA and the UK–eleventhupdate: Resurgence of Cases. Available online at: https://www.ecdc.europa.eu/sites/default/files/documents/covid-19-rapid-risk-assessment-20200810.pdf (accessed May 7, 2021).

